# Molecular Characterization, Tissue Distribution, Subcellular Localization and Actin-Sequestering Function of a Thymosin Protein from Silkworm

**DOI:** 10.1371/journal.pone.0031040

**Published:** 2012-02-22

**Authors:** Wenping Zhang, Changrong Zhang, Zhengbing Lv, Dailing Fang, Dan Wang, Zuoming Nie, Wei Yu, Hanglian Lan, Caiying Jiang, Yaozhou Zhang

**Affiliations:** 1 Institute of Biochemistry, College of Life Science, Zhejiang Sci-Tech University, Hangzhou, China; 2 College of Life Science, Zhejiang University, Hangzhou, China; University of South Florida College of Medicine, United States of America

## Abstract

We identified a novel gene encoding a *Bombyx mori* thymosin (BmTHY) protein from a *cDNA* library of silkworm pupae, which has an open reading frame (ORF) of 399 bp encoding 132 amino acids. It was found by bioinformatics that *BmTHY* gene consisted of three exons and two introns and BmTHY was highly homologous to thymosin betas (Tβ). BmTHY has a conserved motif LKHTET with only one amino acid difference from LKKTET, which is involved in Tβ binding to actin. A His-tagged BmTHY fusion protein (rBmTHY) with a molecular weight of approximately 18.4 kDa was expressed and purified to homogeneity. The purified fusion protein was used to produce anti-rBmTHY polyclonal antibodies in a New Zealand rabbit. Subcellular localization revealed that BmTHY can be found in both Bm5 cell (a silkworm ovary cell line) nucleus and cytoplasm but is primarily located in the nucleus. Western blotting and real-time RT-PCR showed that during silkworm developmental stages, BmTHY expression levels are highest in moth, followed by instar larvae, and are lowest in pupa and egg. BmTHY mRNA was universally distributed in most of fifth-instar larvae tissues (except testis). However, BmTHY was expressed in the head, ovary and epidermis during the larvae stage. BmTHY formed complexes with actin monomer, inhibited actin polymerization and cross-linked to actin. All the results indicated BmTHY might be an actin-sequestering protein and participate in silkworm development.

## Introduction

Thymosins, one family of thymic hormones, play a crucial role in the development and maintenance of a competent immune system, especially in the differentiation and maturation of T-lymphocytes [1], [2], [3], [4]. Thymosins (THY) were originally isolated and purified from calf thymus by Goldstein and White in 1966 [5]. Thymosins belong to a superfamily of small molecular proteins and are divided into 3 main groups (α, β, and γ) based on their isoelectric points [6], [7]. Thymosins exist in many different animal species and are highly conserved and contain one or two THY domains comprised by 37 amino acids. Many studies on the structure and function of thymosin isoforms isolated from vertebrates, such as thymosin alfas (Tα) and Tβ, have been reported. Some of them have been developed into pharmacological agents and widely used in clinical. For example,thymosin-α1 (Tα1),a 28-amino acid polypeptide, is a potent inducer of helper T-cell activity and lymphokine production [8], [9], [10] and is used clinically in certain immunological diseases [11], [12], [13]. Both thymosin β4 (Tβ4) and β10 are G-actin binding proteins [14], [15] and in this manner are believed to play important roles in maintaining cytoskeletal functions, such as cell locomotion and cell spreading, for which actin is a crucial component [16]. Tβ4 also acts as an anti-inflammatory agent, promotes corneal wound healing, and is important in tumor metastasis [17], [18].

However, few studies on thymosin from lepidoptera animal have been reported. There are only 18 related proteins which contain THY conservative structural domain registered in NCBI [19], [20]. These proteins exist in *Drosophila melanogaster, Aedes aegypti, Bombyx mori* and other species, and are highly homologous to Tβ. For instance, the homology of the THY related protein from *Drosophila* to Tβ is 70%. Both of them have a WASP homeodomain [21]. Up to date, no physical or chemical information on the thymosin protein from silkworm is available.

In this study, we identified a silkworm thymosin complementary DNA (*cDNA*; *BmTHY*) from a pupal *cDNA* library (GenBank Accession: FJ602790). Using bioinformatics analysis, we have determined the evolutionary relationship and degree of conservation between BmTHY and other thymosin orthologs. We have also used immunohistochemistry, real-time RT-PCR, and Western blotting analyses to determine its subcellular localization in Bm5 cells (a silkworm ovary cell line), expression patterns in different silkworm developmental stages, and tissue distribution in the fifth instar larvae. Actin sequestering properties of BmTHY were evaluated by nondenaturing gel electrophoresis (NPAGE), sedimentation of polymerization and cross-linking of BmTHY to G-actin.The results provide a solid foundation on which to base further research on the developmental and functional roles played by thymosin in silkworm.

## Materials and Methods

### Materials

The *Escherichia coli* strains TG1 and BL21(DE3) were grown at 37°C in LB medium, pH 7.5 (5 g of yeast extract, 10 g of tryptone and 10 g of NaCl per liter). Bm5 cells [22], a silkworm ovary cell line, were cultured in TC-100 medium (Sigma) supplemented with 10% (v/v) fetal calf serum (FCS, Gibco BRL) at 27°C. The New Zealand white rabbits and BALB/c mice were provided by Zhejiang Academy of Medical Sciences. The animal experiments in this project were conducted according to a protocol approved by the Institutional Animal Care and Use Committee (IACUC) of Institute of Laboratory Animal Sciences, Chinese Academy of Medical Science (The approved No.: N-07-6001). The protocol conforms to internationally accepted guidelines of the animal welfare. The *Bombyx mori* strain used in this study is Qingsong×Haoyue. Silkworms were reared on mulberry leaves under standard conditions. The head, fatty body, intestine, Malpighian tubules, silk gland, skin, trachea and ovary from the fifth instar larvae were dissected, frozen immediately in liquid nitrogen, and stored at −80°C. Nascent eggs, day-5 fifth instar larvae with mulberry leaves removed from the gut, pupae (3 days after pupation), and moths were also frozen in liquid nitrogen and stored at −80°C. Rabbit skeletal muscle actin (>95% pure) and G-actin buffer were obtained from Cytoskeleton Inc.USA. Tβ4 was purchased from Prospec Ltd. Reagents for polyacrylamide electrophoresis, such as acrylamide, bis-acrylamide, tricine, ammoniumpersulfate, and TEMED were purchased from Sigma (USA).

### Bioinformatics Analysis

A *cDNA* sequence encoding the BmTHY protein was obtained from a *cDNA* library of the silkworm pupa constructed in our laboratory [23]. All new data about the cDNA has been deposited in GenBank (GenBank Accession No. FJ602790).The characteristics of the gene were analyzed using DNAstar software (DNASTAR, Inc., USA). Analysis of the similarity of nucleotide and protein sequences was performed using the BLAST algorithm from NCBI (http://www.ncbi.nlm.nih.gov/). The orthologous sequences used for multiple sequence alignments were obtained from NCBI. Multiple sequence alignment was performed with the biosoftware BioEdit (Tom Hall, http://www.mbio.ncsu.edu/BioEdit/bioedit.html). The three-dimensional models for BmTHY protein was built using 3D-JIGSAW software (http://bmm.cancerresearchuk.org/~3djigsaw/).The characteristics of the protein domain and its function were analysed using ExPASy software (http://us.expasy.org/tools).

### Construction of Recombinant Plasmids

We used pHelix-BmTHY constructed by our laboratory [23] as a template to amplify *BmTHY* ORF by PCR, with primers complementary to the flanking sequences of the ORF with *Eco*RI and *Xho*I recognition sites as follows:

P_1_:5′- CGCGAATTCATGGCCTGCTCC -3′;

P_2_:5′- CCGCTCGAGTCAAGCTGATTTCTCTT -3′;

The PCR products were purified after electrophoresis on 1% agarose gel using the PCR Rapid Purification Kit (BioDev-Tech, China).After digestion with *Eco*RI and *Xho*I, the purified PCR products were subcloned into the expression vector pET-28a (Novagen, Darmstadt, Germany) using T4 DNA ligase (Promega, USA) and transformed into *E. coli* TG1 cells (maintained in our laboratory) for screening purposes. A positive colony with *BmTHY* gene in the plasmid was identified by double digestion of the plasmid, followed by analysis on 1% agarose gel electrophoresis and was subsequently verified by DNA sequencing.

### Expression, Purification, and Antibody Preparation of Recombinant BmTHY

The recombinant plasmid pET-28a- BmTHY was transformed into *E. coli* BL21 (DE3) competent cells, which were incubated at 37°C in liquid LB culture media containing 50 µg/mL kanamycin. Expression of the Histag fusion protein was induced at an *A*600 of 0.6 followed by adding IPTG (isopropylthio-*β*-D-galactoside) to a final concentration of 1 mM before another 5-hour incubation. A 5 mL Ni^2+^-SephadexTM G-25 Superfine column (Amersham) was used to purify the expressed recombinant BmTHY (rBmTHY) protein, as instructed by the manufacturer. The concentrated rBmTHY was further purified by reversed-phase FPLC with acetonitrile linear gradient from 5 to 95% in 20 min with the flow rate of 1.7 ml/min and analyzed by the Q Trap LC/MS/MS System (Applied Biosystems, Foster City, CA) to determine its molecular weight. The presence and purity of rBmTHY were evaluated by 12% SDS-PAGE and quantified by the Bradford method [24].

Polyclonal antibody was prepared by immunizing New Zealand White rabbits using purified rBmTHY as antigen [25]. Subsequently, 100 mg of rBmTHY (equal to about 200 ml of the antigen/adjuvant mix) was injected into each of 8–10 subcutaneous sites at the back of the rabbit. In total, 4 times immunizations were done at one-week intervals. For the first time, Freund's Complete Adjuvant was used, and for the other three times, Freund's Incomplete Adjuvant was used. Serum was collected 10 days after the last boost, and was purified using HiTrap Protein A HP (Amersham, Hemel Hempstead, UK) following the manufacturer's instructions. Prior to being loaded, 1 ml HiTrap Protein A HP column was equilibrated with Binding buffer (20 mM sodium phosphate, pH 7.0), and then loaded with anti-serum and washed with 5 column volumes of Binding buffer; anti-BmTHY IgG was eluted with Elution buffer (0.1 M glycine-HCl, pH 3.0) to yield the final fractions. After filtering the IgG through Ultracel PLCHK (Millipore, Billerica,MA), the purified anti- BmTHY IgG was stored in 50% glycerol at −80°C.Indirect ELISA was used to detect the titer of antibody, with negative rabbit sera as control. Western blotting was used to evaluate the specificity of polyclonal antibodies with rBmTHY.

### Protein Extraction

Eggs, the fifth instar larvae, pupae, moths and tissues isolated from the fifth instar larvae were collected and ground to powder in liquid nitrogen followed by suspending in buffer M (50 mM Tris-Cl, pH 8.0; 0.15 M NaCl; 5 mM EDTA; 0.5% NP-40; 1 mM dithiothreitol; 5 mg/mL sodium deoxycholate;100 mg/L PMSF; 5 µg/mL Aprotin (Sigma)), then incubating for 30 minutes on ice. The homogenates were centrifuged at 12 000×g for 15 minutes at 4°C. Protein concentrations were quantitated by the method of Bradford, in which BSA was used as the protein standard [24].

### Western Blotting

Protein samples were equalized and electrophoresed by 10% SDS-PAGE and electrotransfered to polyvinylidene difluoride (PVDF) membranes by semi-dry method with constant current of 2 mA/cm2 for 90 min. The membranes were blocked with 5% skim milk in TBS (pH 7.5). Following incubation with purified anti-BmTHY IgG, membranes were washed and incubated with HRP-labeled anti-rabbit IgG (DingGuo, Beijing, China). Membranes were washed three times with TBS, then washed three times with ddH_2_O, and scanned using the Odyssey Infrared Imaging System (LI-COR, Lincoln, NE) at 700 nm.

### RNA Extraction and Real-Time RT-PCR

Total RNA was extracted from different developmental stages of silkworm and tissues of the fifth instar larvae using Trizol reagent (Invitrogen) according to the manufacturer's instructions. The purity of extracted RNA was determined by UV spectrophotometer. Ratios of UV 260/280 were between 1.8 and 2.1 for all RNA samples analyzed. The concentration of total RNA was determined by measuring the absorbance at 260 nm using SPECTRA max PLUS384 (Molecular Devices). RT-PCR primers were designed using the Primer Select program of the DNA STAR software.

A pair of primers was also designed to amplify 18S ribosomal RNA (rRNA), which was used as an internal control. The primer pairs were as follows:

-(BmTHY) Forward primer, 5′-TGACACTCCCTCCCTGAAAGA-3′, Reverse 5′-CTCCTGGGTCTTCTCAGTGGC-3′;

-(18S rRNA) Forward primer,5′-CGATCCGCCGACGTTACTACA-3′, Reverse 5′-GTCCGGGCCTGGTGAGATTT-3′.

SuperScript III Platinum SYBR Green One-Step RT-PCR Kit with ROX (Invitrogen) was used. 15 µL reaction mixtures contained 7.5 µL 2× SYBR-Green Reaction mixed with Rox, 0.3 µL SuperScript III RT/platinumTaq mix, 1.2 µL 1 µM forward and reverse primers, respectively, 1 µL total RNA and 3.8 µL DEPC-water. RT-PCR was performed by ABI Prism 7300 Sequence Detection System (Applied Bio systems) under the following conditions: an initial cycle at 50°C for 3 minutes, one cycle at 95°C for 5 minutes, followed by 40 cycles of 95°C for 15 seconds, 59°C for 15 seconds, and 72°C for 30 seconds. Each reaction was performed in triplicate in 96-well plates, with the endogenous 18S rRNA control. Dissociation curves were performed to check for the presence of nonspecific dsDNA SYBR Green hybrids, such as primer dimers. Data analysis was performed using ABI Prism 7300 SDS Software V1.3.1 (Applied Biosystems, USA). Expression levels of the target genes were normalized against the expression level of the 18S rRNA gene. The relative expression level was calculated using 2^−ΔΔCT^, where ΔCT = CT_(target gene)_−CT_(18S rRNA)_, ΔΔCT = ΔCT_(target gene)_−ΔCT_(maximum)_ [32].

### Subcellular Localization of BmTHY by Immunofluorescence

Bm5 cells were seeded in dishes for confocal microscopy, and cultured overnight, washed for 10 min three times in PBS, and fixed (PBS, pH 7.2, 4% poly-formaldehyde, 0.1% Triton X-100) at room temperature for 15 min. The fixed cells were blocked with 3% BSA at room temperature for 2 h followed by three 10-min washes in PBST (0.05% Tween-20 in PBS). Cells were then incubated with purified anti-rBmTHY polyclonal antibody (diluted 1∶200 in blocking buffer) at 4°C overnight; cells were simultaneously incubated with negative serum as a control. The negative serum was obtained from the rabbit before immunizing with antigen. After three 10-min washes in PBST, cells were incubated with Cy3-labeled goat anti-rabbit IgG (diluted 1∶500; Promega) at 37°C for 2 h and were then washed twice for 10 min in PBST. Cells were then incubated with 4′-6-diamidino-2-phenylindole (1 g/ml in PBS) at room temperature for 10 min. Cells were washed three times with PBST (10 min each), and analyzed with a Nikon ECLIPSE TE2000-E Confocal Microscope (Nikon, Tokyo, Japan) with image analysis software EZ-C1.

### Binding of BmTHY to Actin in Nondenaturing Gels

Nondenaturing polyacrylamide gel electrophoresis (NPAGE) was performed in gels containing 25 mM Tris, 194 mM glycine, 0.2 mM ATP, 0.1% Triton X-100, 7.5% acrylamide, 0.2% N,N′-methylenebisacrylamide, 0.03% N,N,N′JV′-tetramethylethylenediamine, and 0.1% ammonium persulfate. The electrode buffer contained 25 mM Tris, 194 mM glycine, and 0.2 mM ATP. Gels were prerun at 20–25 V/cm for 1 h in a 4°C coldbox; the electrode buffer was poured off and replaced, and samples were then loaded and run at 20–25 V/cm at 4°C [26].

0.6–1.2 µg of Tβ4 or BmTHY were mixed with 2.0 µg of rabbit muscle actin monomers in 12 µLof G-buffer containing 0.5 mM ATP, **0.5** mM β-mercaptoethanol,0.2 mM KCl, 10 mM Tris-HC1, pH 7.5. After a 10-min incubation at room temperature, 3 µL of glycerol with a trace of bromphenol blue was added, and the samples were electrophoresed at 4°C at 20–25 V/cm until the bromphenol blue dye front ran out.The proteins were stained with Coomassie Blue to get electrophoresis pattern.

### Sedimentation Assays for Actin Polymerization

Sedimentation Assays for Actin Polymerization was performed using a Beckman Airfuge with fixed-angle rotor as previously described [27]. G-actin was added as a 11.5 µM solution in G-buffer (0.2 mM ATP, 0.2 mM CaCl_2_,0.2 mM dithiothreitol, 0.2 mM NaN3, **3** mM Tris-HCl, pH 7.6); Tβ4 or BmTHY were added as 10 µM solutions in KCl buffer (137 mM KCl, 12 mM NaHC0_3_, 5 mM MgCl_2_, 10 mM HEPES, 0.36 mM NaH_2_P0_4_, pH 7.1). For each assay, 100 µL of actin was mixed with the specified volume of peptide solution, and the final volume was brought to 200 µL with KCl buffer. After mixing, the actin was allowed to polymerize for 15 min at 25°C before centrifuging at 100,000× *g* for 30 min. The supernatants were pipetted off, and each pellet was solubilized in 50 µL of SDS sample buffer. Equal aliquots were analyzed by SDS-PAGE on a 10% gel.

### Cross-linking of BmTHY to G-actin

Cross-linking of BmTHY or Tβ4 to G-actin was analyzed according to the method described by Safer [27].G-actin (23 µM) 3 mM triethanolamine-HC1,0.2 mM CaC1_2_, 0.2 mM ATP, 0.2 mM NaN_3_, pH 7.5, was incubated at 4°C for 45 min with either BmTHY or Tβ4 at different molar ratio. Aliquots of 10 µL were then mixed with 12.2 µL of 5.4 mM l-ethyl-3-(3-dimethylaminopropyl) carbodimidie in 0.1 M MES, pH 6.5, and incubated for 2 h at 25°C. Equal aliquots were taken up into SDS sample buffer and analyzed by SDS-PAGE on a 10% gel under reducing conditions.

## Results

### Bioinformatics Analysis of BmTHY

**Figure 1:**
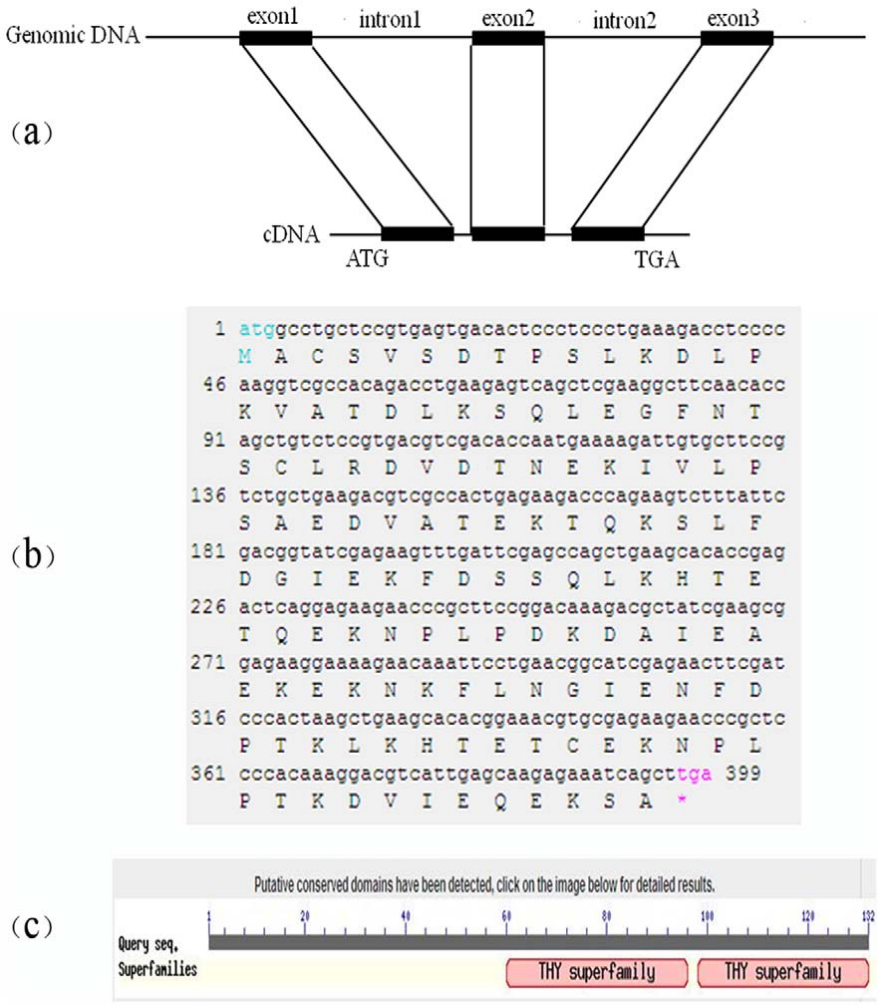
Profile of silkworm *BmTHY* gene. (a) Schematic representation of *BmTHY* gene. (b) ORF sequence and predicted amino acid sequence of *BmTHY* gene. (c) BmTHY contains two intact THY domains.

The *BmTHY cDNA* sequence (GenBank accession number FJ602790) was identified from a *cDNA* library of the silkworm pupa constructed in our laboratory [23]. It consists of 1,656 bp and contains an ORF of 399 bp encoding a protein of 132 amino acids. This gene, *BmTHY*, contains two introns and three exons (Fig. 1a). The BmTHY *cDNA* sequence and predicted amino acid sequence are shown in Fig. 1b. The predicted protein contains two complete THY conserved domains (Fig. 1c) and has a predicted molecular weight of 14.74 kDa, with a theoretical pI of 4.83. BmTHY is an acidic protein.

**Figure 2:**
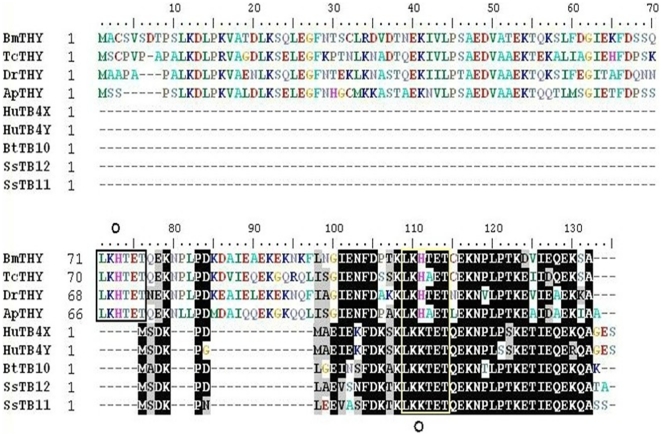
Multiple amino acid sequence alignment of BmTHY with homologous proteins. The identical residues were shaded in black, while the similar residues were shaded in gray, Ruler shows the number and position of residue. BmTHY, TcTHY, DrTHY and ApTHY represent thymosin superfamily protein of *Bombyx Mori, Tribolium castaneum, Drosophila melanogaster, Apis mellifera* respectly; HuTB4X:Tβ4(X-Chromosome,*Homosapiens*);HuTB4Y:Tβ4(Y-chromosome,*Homosapiens*);BtTB10:Tβ10(*Bos Taurus*);SsTB12:Tβ12(*Salmo salar*);SsTB11:Tβ11(*Salmo salar*).

**Figure 3:**
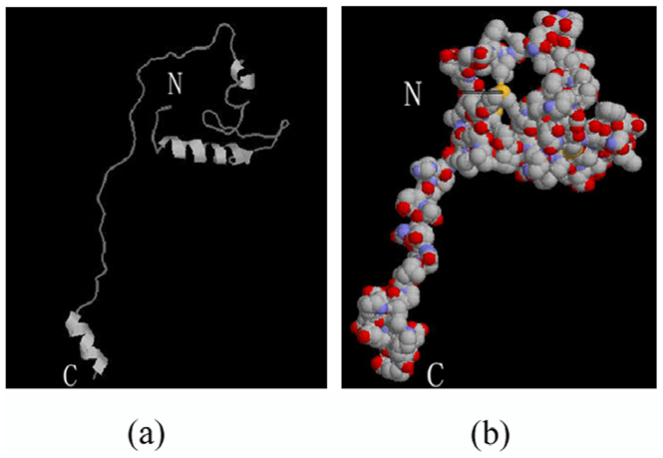
The tertiary structure of BmTHY. (a)The tertiary structure of BmTHY(strands); (b)The tertiary structure of BmTHY(Molecular Surface).

By multiple sequence alignment, it was found that BmTHY had a high degree of homology to Tβ proteins, especially in the conserved domains. For example,the amino acid sequence similarities between two conserved domains from BmTHY and human Tβ protein are 74% and 81% respectively. The conserved domains from BmTHY and insect thymosin superfamily protein are of high sequence similarity, especially in the N-terminus (shown in Fig. 2).The six peptide motif ‘LKKTET’ of Tβ family proteins is very conservative and plays a key role in the process of Tβ binding to actin [14].The relatively conserved sequence also exists in BmTHY and a six peptide motif ‘LKHTET’ of BmTHY is shown in Figure 2, with only one amino acid difference from ‘LKKTET’. Structure predictions indicate that there are an N-terminus α-helix and a C-terminus α-helix in the three-dimensional conformation of BmTHY (Fig. 3.). The structure of intermediate sequence is relatively simple but its N-terminus is complicate.

### Expression and Purification of Recombinant BmTHY

**Figure 4:**
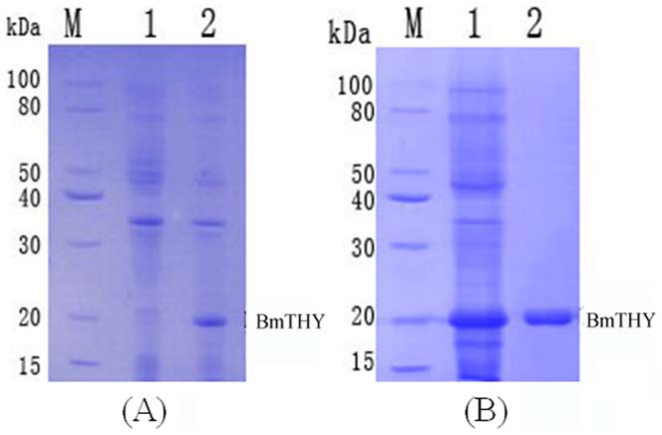
Expression and purification of the His-tag-BmTHY fusion protein. Samples were resolved by 12% SDS-polyacrylamide gel electrophoresis under reducing conditions. A: Expression of fusion protein in Rosetta (DE3); M: protein molecular weight marker (low); 1: Rossetta (pET-28a-BmTHY) without induction; 2: Rossetta (pET-28a-BmTHY) after induction; B: Purification of the His-tag fusion protein in Rosetta (DE3); M: protein molecular weight marker (low); 1: supernatant of *E.coli* Rosetta/pET-28a-BmTHY induced by IPTG after supersonic treatment; 2: purified fusion protein expressed in *E.coli* Rosetta.

**Figure 5:**
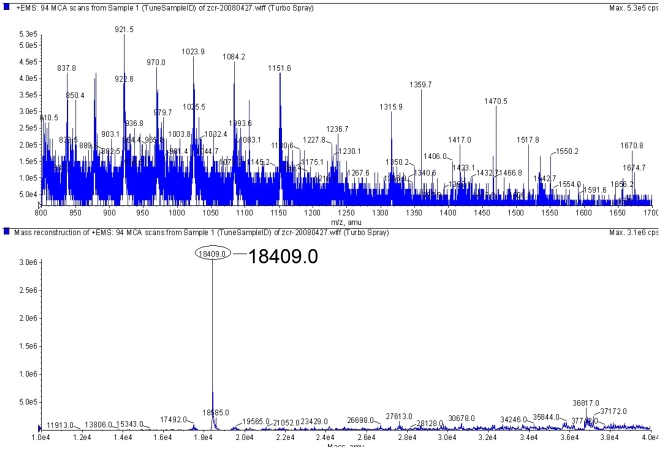
Analysis of the His-tag-BmTHY fusion protein by MS.

We confirmed expression of recombinant BmTHY in *E. coli* by SDS-PAGE. The result of 12% SDS-PAGE on recombinant BmTHY showed a protein of expected size in recombinant bacteria (Fig. 4A). Histagged rBmTHY could be purified by HiTrap Chelating HP (Fig. 4B). The predicted molecular weight of BmTHY is 14.74 kDa, and the molecular weight of the His label is 3.56 kDa. We confirmed the molecular weight of the fusion protein by mass spectrometry, which indicated a molecular weight of 18.4 kD (Fig. 5), matching the theoretical value of 18.3 kD (14.74 kD+3.56 kD) well. The results indicated that rBmTHY was expressed successfully.

### Titer Analysis and Specificity Detection of the Polyclonal Antibodies

**Figure 6:**
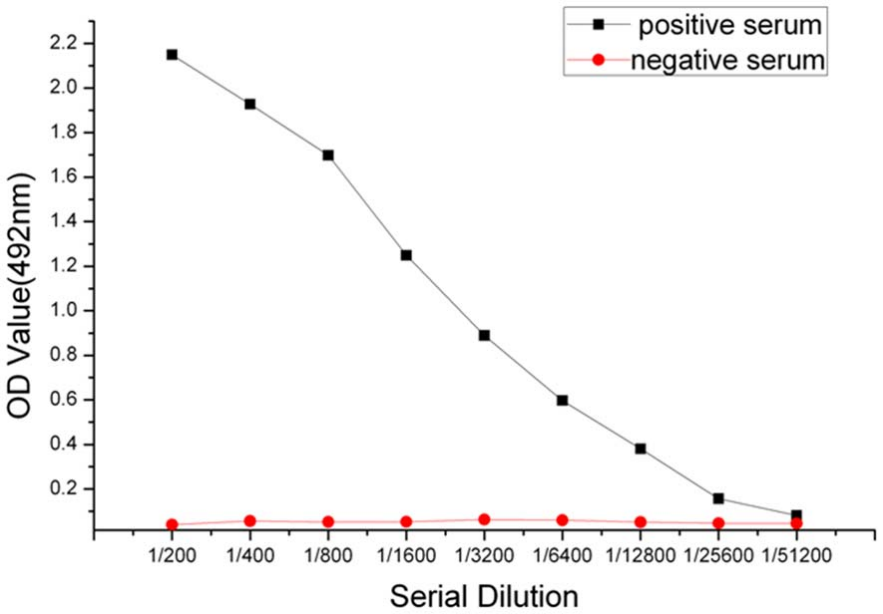
Determine of polyclonal antibody titer by ELISA. ELISA was used to determine the following ratio for antibodies against rBmTHY: positive serum extinction value/negative serum extinction value (P/N)≥2.1 was positive; 1.5≤P/N<2.1 was suspicious expression; P/N<1.5 was negative. From this, the titer of the antibodies was greater than 1∶25600 at a concentration of 10 µg/mL.

**Figure 7:**
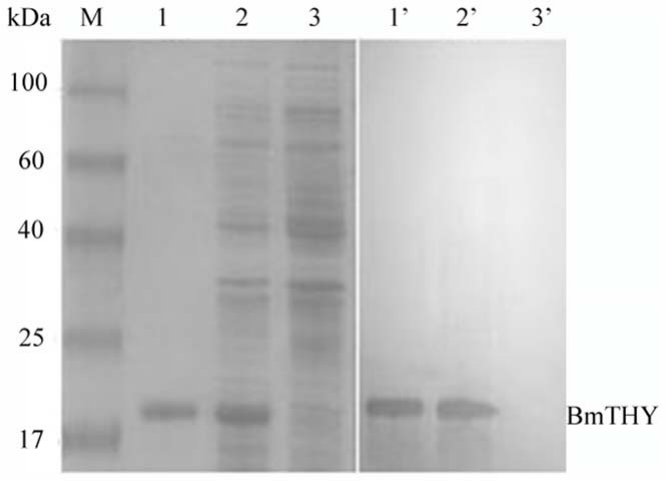
Western blotting analysis of the His-tag-BmTHY fusion protein expression. Samples were resolved by 12% SDS PAGE under reducing conditions (M,1,2,3 SDS-PAGE; 1′, 2′,3′ Western blotting). M: protein molecular weight marker (low); 1: purified fusion protein expressed in *E.coli* Rosetta; 2: supernatant of *E.coli* Rosetta/pET-28a-BmTHY induced by IPTG after supersonic treatment; 3: *E.coli* Rosetta/pET-28a. Arrow indicates the fragment of the His-tag fusion BmTHY.

We used ELISA, after obtaining all extinction values, to determine the following ratio for antibodies against rBmTHY: positive serum extinction value/negative serum extinction value (P/N)≥2.1 was positive; 1.5≤P/N<2.1 was suspicious expression; P/N<1.5 was negative. From this, the titer of the antibodies was greater than 1∶25600 at a concentration of 10 µg/mL, which had met requirement for next experiments (Fig. 6). The specificity of anti-rBmTHY polyclonal antibodies was determined by Western blotting. The anti-rBmTHY rabbit serum generated using the purified recombinant protein strongly reacted with the 18.4-kDa rBmTHY expressed in induced *E. coli* extracts, while no signal was detected in uninduced extracts (Fig. 7). These results illustrated high specificity of the polyclonal antibodies.

### Transcription and Expression Levels of BmTHY at Various Silkworm Developmental Stages

**Figure 8:**
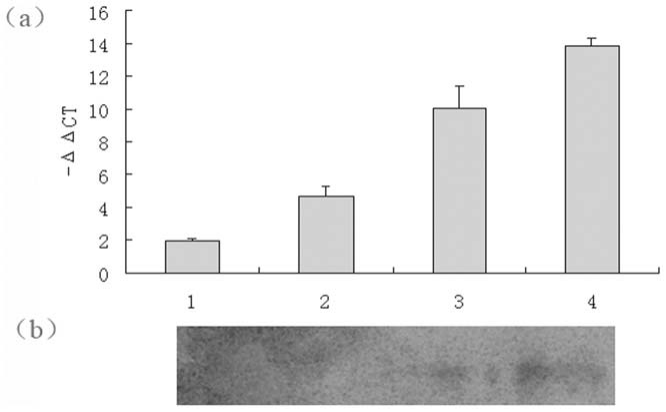
Transcription and expression level of BmTHY in different development stages of *Bombyx mori*. (a) Analysis of BmTHY expression was performed by RT-PCR. Relative BmTHY expression was determined in relation to the corresponding BmTHY expression level in the silkworm moth: ΔΔC_T_ (stage) = ΔC_T_ (stage)−ΔC_T_ (egg); (b) Western blotting analysis of the expression levels of of BmTHY in different development stages. 1,egg;2,pupa; 3,larva;4,moth.

We performed Real-Time RT-PCR analysis on mRNA to determine BmTHY transcription levels at four different silkworm developmental stages like eggs, the fifth instar larvae, pupae, and moths. From the melting curves (data not shown), we established, there were no overt primer dimers, indicating good primer specificity. The amplification curves (data not shown) showed good reproducibility. The PCR results indicated an obvious difference in BmTHY transcription levels between the four silkworm developmental stages. BmTHY mRNA was highest in moth, lower in instar larva, pupae, and lowest in egg (Fig. 8a).

In order to determine the protein expression levels of BmTHY at silkworm various developmental stages, we extracted total protein from the egg, fifth instar larvae, pupa, and moth. Western blotting analyses were performed on protein extracts to determine BmTHY expression levels. The highest amount of expressed BmTHY was in moth, decreasing in the fifth instar larvae;no BmTHY was expressed in egg or pupa (Fig. 8b).

### Transcription and Expression Level of BmTHY in Different Fifth-Instar Larva Tissues

**Figure 9:**
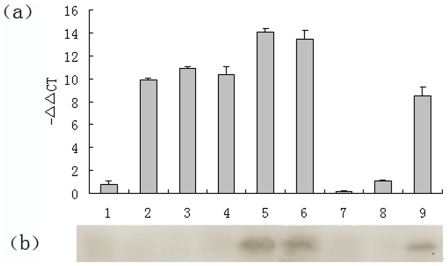
Transcription and expression level of BmTHY in different tissues of fifth-instar larva of *Bombyx mori*. (a) Transcription level of BmTHY in different tissues of fifth-instar larva of *Bombyx mori*. Total RNA from the silk gland, spiracle,midgut, Malpighian tubule, head, ovary, testis,fatty body, and epidermis of fifth-instar larva was used as a template for RT-PCR. Relative BmTHY expression was determined in relation to the corresponding BmTHY expression level in testis: ΔΔC_T_ (tissue) = ΔC_T_ (tissue)−ΔC_T_ (testis); (b) Western blotting analysis of the expression levels of of BmTHY in different development stages. 1:Silk gland;2:Spiracle;3:Midgut; 4: Malpighian tubule;5:Head;6:Ovary; 7: Testis;8:Fatty body;9: Epidermises.

In order to determine transcription levels of BmTHY in the head, Malpighian tubule,epidermis, spiracle, silk gland, testis,ovary, gut, and fatty body of the fifth-instar larvae, total RNA was isolated from these tissues for RT-PCR. From the amplification curves and melting curves(data not shown), we determined that there was excellent reproducibility and no overt primer dimers formed during amplification, indicating good specificity for the primer pair. BmTHY mRNA was universally distributed in the examined tissues, with the transcription level of BmTHY highest in the head and ovary,and lower in the gut, spiracle, Malpighian tubule, epidermis, fatty body, silk gland and testis (Fig. 9a).

To elucidate the expression of BmTHY protein in the head, Malpighian tubule, epidermis, spiracle, silk gland, ovary, testis, gut, and fatty body of fifth-instar larvae, we extracted protein from these tissues, and performed Western blotting analysis to determine the amount of BmTHY in each. The result of immunoblots on protein extracts revealed that anti-BmTHY serum reacted with a 18.4-kDa protein in extracts isolated from head, ovary and epidermis, but no signal was detected in extracts isolated from silk gland, spiracle, gut, Malpighian tubule, testis and fatty body (Fig. 9b).

### Subcellular Localization of BmTHY

**Figure 10:**
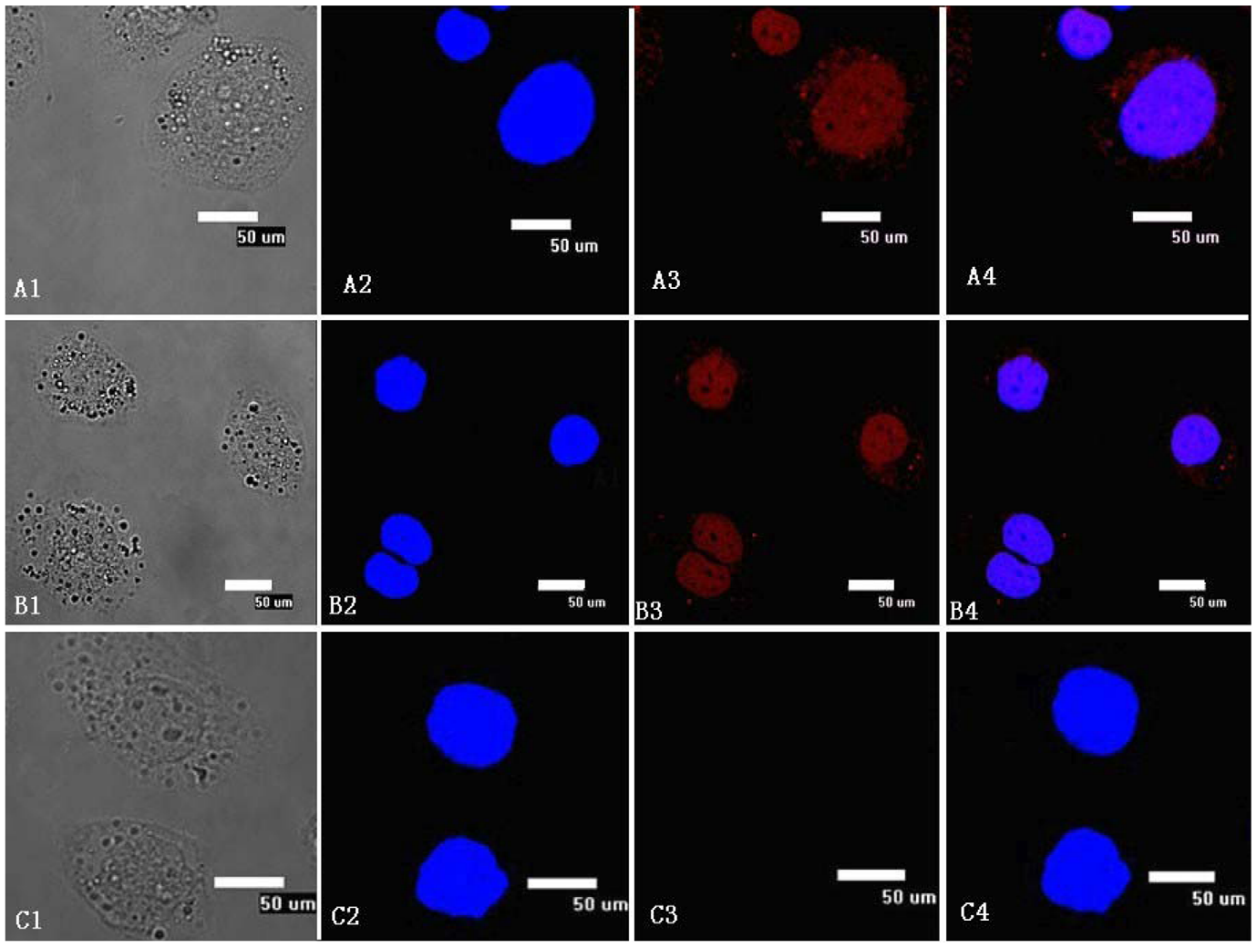
Subcellular localization of BmTHY in Bm5 cells by immunofluorescence. A1–A4,B1–B4: Experimental group, using anti-BmTHY polyclonal antibody;C1-C4: negative control group, using negative rabbit serum;A1,B1,C1: Cells under transmitted light. A2,B2,C2: DAPI staining;A3,B3,C3: BmTHY subcellular localization as indicated by Cy3-labeled secondary antibody;A4,B4,C4: merged image.

The treated cells were examined under a Nikon ECLIPSE TE2000-E Confocal Microscope,and images were analyzed using EZ-C1software. Cy3-labeled goat anti-rabbit IgG emitted red fluorescence when stimulated with light having a wavelength of 550 nm, and DAPI-stained nuclei emitted red fluorescence when stimulated with light having a wavelength of 353 nm. The results indicated that BmTHY was mainly located in nucleus, although it was rarely present in the cytoplasm (Fig. 10).

### Actin Sequestering Properties of BmTHY

**Figure 11:**
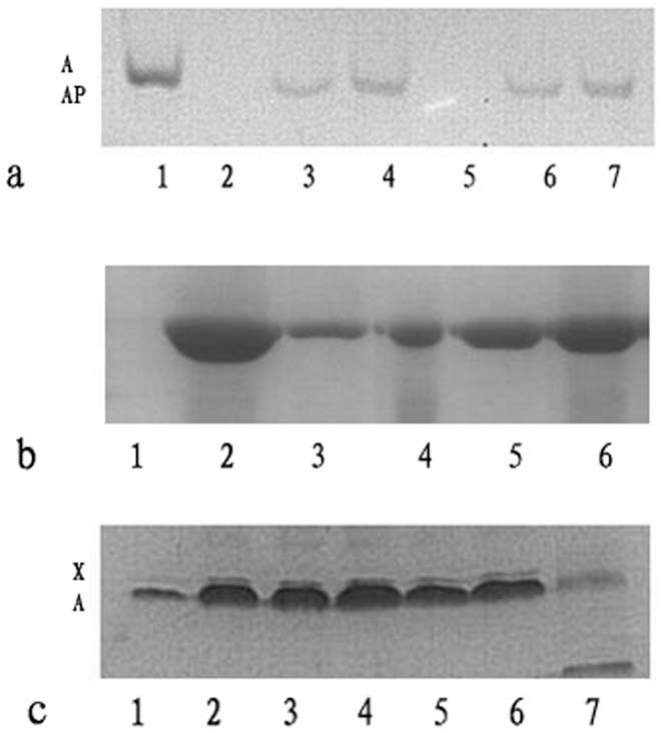
Electrophoretic analysis of the interaction of actin with BmTHY. a,Effect of BmTHY and Tβ4 on the mobility of actin in nondenaturing polyacrylamide gel. NPAGE of muscle G-actin plus BmTHY or Tβ4 shows that both BmTHY and Tβ4 shift the mobility of actin in a stoichiometric fashion. Lane 1,2 µg of G-actin ; Lane2 and 5,blank; Lane 3, 0.6 µg of Tβ4 and 2 µg of G-actin ; Lane 4, 1.2 µg of Tβ4 and 2 µg of G-actin ; Lane 6, 0.6 µg of BmTHY and 2 µg of G-actin ; Lane 7, 1.2 µg of BmTHY and 2 µg of G-actin. b,sedimentation analysis of the effect of BmTHY and Tβ4 on salt-induced actin polymerization. 100 µL of G-actin was mixed with:100 µL of G-buffer (lane 1); 100 µL of KCl-buffer (lane 2); 66 µL of Tβ4 (lane 3); 66 µL of BmTHY (lane 4);33 µL of Tβ4 (lane 5); 33 µL of BmTHY (lane 6);The molar ratio of BmTHY (Tβ4) to actin was 0.57∶1 in lanes 3 and 4 and 0.28∶1 in lanes 5 and 6. The final actin concentration was 5.8 µM in each assay. Each pellet was solubilized in 50 µL of SDS sample buffer. c,cross-linking of BmTHY and Tβ4 to G-actin. G-actin was incubated with either BmTHY (lane 2, 3 and 4) or Tβ4 (lane 5 and 6) plus 1-ethyl-3-(3-dimethylaminopropyl) carbodimide for 2 h at 25°C. The molar ratio of BmTHY (Tβ4) to actin was 1∶5 in lanes 2, 3 and 5 and 1∶9 in lanes 4 and 6. Lane 1 was G-actin. Lane 7 was protein molecular weight marker. Each aliquots were taken up into SDS sample buffer and analyzed by SDS-PAGE on a 10% gel under reducing conditions.

Binding of BmTHY to Actin in Nondenaturing Gels was firstly analyzed. BmTHY and Tβ4 both formed complexes with skeletal muscle G-actin. Fig. 11a shows that the complexes of BmTHY and G-actin migrated more rapidly than G-actin alone, like that of Tβ4 and G-actin. This is consistent with the observation demonstrated by Safer *et al.* [27], [28] The results demonstrated that BmTHY is an actin-binding protein.

Incubation of BmTHY with G-actin inhibited salt-induced polymerization by an equivalent amount as assayed by sedimentation. Fig. 11b (lane 4 and 6) shows that BmTHY decreased the sedimentability of actin under polymerizing conditions. With increasing BmTHY concentrations, less actin was recovered in the pellet after high speed centrifugation. In the positive control group (Fig. 11b, lane 3 and 5), similar inhibition was observed with Tβ4 as described previously [15], [27]. Fig. 11c indicates that both BmTHY and Tβ4 could be covalently linked to actin by adding the cross-linker l-ethyl-3-(3-dimethylaminopropyl) carbodiimidie to a stoichiometric mixture of actin with each peptide. A covalently cross-linked product was formed in both cases (Fig. 11c, lane 2,3,4,5 and 6).The above results indicated that BmTHY is an actin-sequestering protein similar to Tβ4.

## Discussion

Tβ protein family has a relatively conserved motif ‘LKKTET’ involving Tβ binding to actin [29], [30]. Interestingly, through bioinformatics analysis, it is found that BmTHY also has a relatively conserved motif ‘LKHTET’ with only one amino acid difference from ‘LKKTET’. Therefore, we speculated that BmTHY may bind to actin by means of the conserved motif ‘LKHTET’ to play its role. It is reported that proteins homologous to Tβ found in Drosophila may bind to actin in the same way as Tβ4,but exert the same function as Profilin in cell [31]. Profilin plays an important role in many important cellular processes, including membrane trafficking, GTPase signal transduction, transcription regulation, RNA splicing, neurological genesis and differentiation [32], [33], [34], [35], [36].The results of tertiary structure analysis showed that there is an α-helix at both C-terminus and N-terminus of BmTHY. N-terminus structure of BmTHY is more complex than that of Tβ. However both N-terminus sequences of them are different. These different structures from each other may lead to their different function.

In this study, we made some experiments on BmTHY to analyze its characteristics and functions. Recombinant BmTHY protein was expressed highly in *E. coli* and was mostly in the supernatant of *E.coli* Rosetta/pET-28a-BmTHY induced by IPTG after supersonic treatment. Recombinant BmTHY protein was also purified easily by affinity chromatography. The molecular weight of the purified fusion protein was confirmed by mass spectrometry, which indicated a molecular weight of 18.4 kD (Fig. 5), matching the theoretical value of 18.3 kD well. The results verified that recombinant BmTHY was expressed successfully.

Analysis on bioinformatics and actin-sequestering function of BmTHY (Fig. 11) suggests that BmTHY, like Tβ, may well be an actin-binding protein which is involved in cellular activity in that actin represents one of the major cytoskeletal components and participates in cellular processes such as movement and morphogenesis [37]. Silkworm goes through a complete metamorphosis development process, including egg, larva, pupa, and moth, four developmental stages that have great differences in morphology, physiological characteristics, and biological function [38], [39]. In the larva and moth stage, more and more actins will polymerize or depolymerize due to morphological changes and physiological peculiarity, which needs more and more actin-binding proteins regulation [39]. Therefore, the transcription and expression level of actin-binding proteins will increase during larva and moth stage. We performed Real-Time RT-PCR analysis on mRNA to determine BmTHY transcription levels at four different silkworm developmental stages. The PCR results indicated BmTHY mRNA was highest in moth, lower in instar larva, pupae, and lowest in egg. Western blotting to determine the expression pattern of BmTHY at different developmental stages of *B. mori* showed that BmTHY was expressed in moth and larva. This discovery suggests that BmTHY might participate in the regulation of morphological changes in silkworm by means of binding to actin. However, no positive signals of western blotting were detected in egg and pupae. One possibility is that the expression of BmTHY in the egg or pupae developmental stages is too little to be detected, or there is no expression in both stages. We are apt to the former because the result of the transcription profile analysis showed that BmTHY mRNA was also found in both pupae and egg by Real-Time RT-PCR.

Because both Real-Time RT-PCR and Western blotting analysis testified BmTHY expressed in the fifth instar larva, we extracted total RNA and proteins from nine fifth-instar larva tissues to further examine transcription and translation of the *BmTHY* gene. The result of RT-PCR revealed that the BmTHY mRNA was universally distributed in most of tissues (except testis) extracted from the fifth-instar larvae. BmTHY transcription level in the tested tissues from high to low in turn is orderly: head>ovary>midgut>spiracle>Malpighian tubule>epidermises>fatty body>silk gland>testis. The result of Western blotting showed that BmTHY was expressed in the head, ovary and epidermis. Why was BmTHY expressed in ovary but testis? We presume that BmTHY might be involved in sex differentiation in the developmental stage of silkworm. Why was BmTHY expressed in head and epidermis? We speculate that BmTHY might participate in cell differentiation and migration because both head and epidermis are important parts of morphological changes in silkworm.

We carried out subcellular localization of BmTHY in Bm5 cell by immunofluorescence. BmTHY was found mostly in the nucleus but was also observed in the cytoplasm. Some actin-binding proteins have been previously reported to localize intracellularly to or shuttle into the nucleus [30], [40], [41], [42]. For example, Huff [40] reported that Tβ4 served as a G-actin sequestering peptide in the nucleus and was specifically translocated into the cell nucleus by an active transport mechanism, requiring an unidentified soluble cytoplasmic factor. Kim demonstrated that nuclear localization of thymosin β15 was a controlled process during kainic acid or staurosporine stimulation [42].Interestingly, the subcellular localization of BmTHY in this study was found to be similar to that of Tβ4. The experimental results suggested that BmTHY might be translocated into the cell nucleus depending on synergistic effects of some cytokines. Analysis on actin sequestering of BmTHY shows that BmTHY not only forms complexes with actin, inhibits salt-induced G-actin polymerization but also covalently links to actin. Considering the experimental results of subcellular localization and actin sequestering of BmTHY together, we speculate as the following : By binding to nuclear actins, BmTHY might be involved in the physiological function of actin, such as RNA splicing, chromosome remolding, and transcriptional regulation [43], [44], [45].

In a word, the presented data indicated that BmTHY might participate in the regulation of morphological changes and physiological function in silkworm development by means of binding to actin.
